# Endogenous Expression of G-CSF in Rat Dorsal Root Ganglion Neurons after Nerve Injury

**DOI:** 10.3390/brainsci11070956

**Published:** 2021-07-20

**Authors:** Chun-Chang Yeh, Chih-Ping Yang, Kuo-Hsing Ma, Jui-Hu Shih, Ching-San Tseng, Yuahn-Sieh Huang

**Affiliations:** 1 Department of Anesthesiology, National Defense Medical Center, Tri-Service General Hospital, Taipei 11490, Taiwan; anes2yeh@gmail.com (C.-C.Y.); ycp810@gmail.com (C.-P.Y.); 2 Department of Anesthesiology, Chi-Mei Medical Center, Tainan 71004, Taiwan; 3 Department of Biology and Anatomy, National Defense Medical Center, Taipei 11490, Taiwan; kuohsing91@yahoo.com.tw (K.-H.M.); tinying1026@gmail.com (C.-S.T.); 4 Department of Pharmacy Practice, Tri-Service General Hospital, Taipei 11490, Taiwan; jtlovehl@gmail.com; 5 School of Pharmacy, National Defense Medical Center, Taipei 11490, Taiwan

**Keywords:** G-CSF, G-CSFR, DRG, neuropathic pain

## Abstract

Granulocyte colony-stimulating factor (G-CSF) has been reported to modulate pain function following nerve injury. However, the expression of endogenous G-CSF in the dorsal root ganglion (DRG) and the response to nerve injury remain unclear. In the present study, we demonstrated that G-CSF and G-CSFR are mainly expressed in both small- and medium-diameter DRG neurons in rats and are responsible for transmitting pain responses. G-CSF and G-CSFR were co-expressed in certain nociceptive DRG neurons. In addition, G-CSF was expressed in satellite glial cells around large-diameter DRG neurons. After sciatic nerve injury, the number of G-CSF-positive DRG neurons was increased in both the ipsilateral and contralateral lesion sites in rats. However, G-CSF expression in satellite glial cells was not affected by nerve injury. To clarify the role of G-CSF in pain, exogenous G-CSF was administered to a rat model of neuropathic pain induced by partial sciatic nerve transaction (PST). Our results indicate that treatment with G-CSF did not attenuate but exacerbated neuropathic pain. In summary, G-CSF may directly activate sensory neurons and contribute to nociceptive signaling.

## 1. Introduction

Neuropathic pain is chronic pain caused by damage to the central or peripheral nervous system. It is characterized by an abnormal or excessively sensitive response to external stimuli [1,2,3]. This condition often impairs patients’ quality of life. At present, neuropathic pain is difficult to treat. Although opioid analgesics are the main clinical drugs used for patients, patients often experience poor clinical outcomes [4,5,6], largely because the exact pathophysiological mechanism of neuropathic pain remains unclear. It is important to elucidate the pathological mechanism of neuropathic pain and to find a way to treat this condition.

Granulocyte colony-stimulating factor (G-CSF) is a 19.6 kDa glycoprotein that is used clinically as a growth factor to induce the differentiation of murine myelomonocytic leukemia cells and as a growth factor to promote the survival, proliferation, and differentiation of neutrophil granulocytes [7]. G-CSF is clinically used in patients with leukopenia and is administered to donors for the collection of hematopoietic progenitor cells prior to transplantation [8,9]. G-CSF is mainly secreted by monocytes [10,11], but it is also expressed in other types of cells, including fibroblasts [12], endothelial cells [13], mesothelial cells [14,15], stromal cells [13], astrocytes [16], nerve cells [17,18], and even tumor cells [19], suggesting that G-CSF may have different regulatory effects on various cells. The nonhematopoietic effects of G-CSF, including its effects on the central nervous system, have also been reported [17,20,21]. In a rat cerebral ischemia model, it was found that G-CSF can protect neurons from ischemia-induced cell death and promote nerve regeneration [21,22]. It has also been reported that in mouse and rat spinal cord injury models, G-CSF can protect neurons and oligodendrocytes from apoptosis [23,24]. In addition, G-CSF may be involved in the regulation of pain [25,26]. G-CSF relieves neuropathic pain caused by spinal cord injury and nerve compression by inhibiting inflammation [24,27,28]. In contrast, another study reported that G-CSF can aggravate neuropathic pain [29]. Although G-CSF is involved in the modulation of pain modality, whether endogenous G-CSF is expressed in the dorsal root ganglion (DRG) and the changes in its expression after nerve injury are still unclear.

In this study, we found that endogenous G-CSF was expressed in the DRG and that G-CSF and G-CSFR were co-expressed in both small- and medium-diameter DRG neurons. G-CSF expression was increased by sciatic nerve injury. Exogenous administration of G-CSF increased pain in a rat model of pathological pain, suggesting that G-CSF can act directly on the DRG and may be involved in the modulation of pathological sensation.

## 2. Materials and Methods

### 2.1. Antibodies and Reagents

The following antibodies were used: goat anti-G-CSF, rabbit anti-G-CSFR (Santa Cruz Biotechnology, Santa Cruz, CA, USA), anti-TRPV1 [BS397] (Abcam, Cambridge, UK), mouse anti-GFAP (DAKO, Glostrup, Denmark), DyLight™ 488-conjugated donkey anti-goat IgG, and DyLight™ 594-conjugated donkey anti-rabbit IgG (Jackson ImmunoResearch Laboratories, West Baltimore Pike, PA, USA). The dye IB4 was obtained from Sigma-Aldrich (St. Louis, MO, USA).

### 2.2. Animals

This study was approved by the Animal Care and Use Committee of the National Defense Medical Center (Taipei, Taiwan; Approval No. IACUC-16-061) and was conducted in accordance with the Guide for the Care and Use of Laboratory Animals published by the National Institutes of Health (Bethesda, MD, USA). Male Wistar rats (BioLASCO, Taipei, Taiwan), weighing 200–250 g, were housed individually with soft bedding on a 12 h night/day cycle and provided free access to food and water at all times. They were housed in a similar environment for 7 days for acclimation before the experiment. All efforts were made to minimize the number of animals used and their suffering. Mechanical allodynia was assessed in the animals, using a dynamic plantar anesthesiometer (DPA), at which point there was no clinical evidence of nerve damage, and on various days (1 day, 7 days, and 14 days) after PST.

### 2.3. Establishment of the Neuropathic Pain Animal Model

Partial sciatic nerve transection (PST) was performed, according to the previously reported protocols [30]. All rats then underwent either PST or sham operation. In the PST rats, the left sciatic nerve was exposed at the mid-thigh level, and a prolene 7–0 ligature was placed through the midpoint of the nerve just cranially to the branch running to the musculus biceps femoris. Half of the diameter of the nerve was transected in a ventrocranial direction up to the ligature; the ligature was then removed. In the sham-operated rats, the nerve was exposed, and then the wound was closed with sutures.

### 2.4. Behavioral Testing

Mechanical allodynia was assessed using a dynamic plantar aesthesiometer (DPA) (Ugo Basile, Comerio, Italy), which is an automated version of the von Frey filament test that does not induce tissue damage [31,32]. According to the Kyoto protocol of the International Association for the Study of Pain, Basic Pain Terminology, DPA produces non-noxious tactile stimuli [33]. Each rat was placed in an individual plastic cage (25 cm long × 10 cm wide × 14 cm high) with a wire mesh floor and was acclimatized to the cage for 15 min before each test session. Both hind paw withdrawal responses were elicited by applying an increasing force, using a blunt-end metal filament (0.5 mm in diameter) focused on the area of the sural nerve on the palmar surface of the left ipsilateral or right contralateral hind paw. The force was increased from 1 to 50 g in steps of 1 g over 20 s and was then held at 50 g for an additional 10 s; the rate of the increase in force was 2.5 g/s. The threshold was recorded as the force that elicited the hind paw withdrawal reflex (the mean of three measurements performed at 1 min intervals).

### 2.5. G-CSF Administration

The rats were randomly divided into 7 groups (6 rats in each group): vehicle (normal saline solution), G-CSF (10 μg/kg), G-CSF (100 μg/kg), the sham+vehicle group, PST+vehicle, PST+G-CSF (10 μg/kg), and PST+G-CSF (100 μg/kg). G-CSF was administered by intravenous (i.v.) tail vein injection once a day for 2 days before PST.

### 2.6. Tissue Preparation and Sectioning

The rats were anesthetized by intraperitoneal injection of chloral hydrate, perfused with saline using a peristaltic circulation pump (Cole-Parmer Instrument Company, Vernon Hills, IL, USA), and then fixed with 4% paraformaldehyde. The L4/5 DRG of each rat was removed, postfixed with 4% paraformaldehyde, and then sequentially placed in 20% and 30% sucrose solution for dehydration and cryoprotection. After the tissue samples sank to the bottom, they were embedded in an optimum cutting temperature freezing medium (Thermo Electron Co., Waltham, MA, USA) and then sectioned with a cryostat (Leica, Wetzlar, Germany). The thickness of the slices was 16 μm.

### 2.7. Immunohistochemistry

Free-floating sections were washed with a phosphate-buffered saline (PBS) and then treated with 1% hydrogen peroxide (H_2_O_2_) for 30 min to block endogenous peroxidase activity. After washing with PBS, the sections were blocked with 5% normal horse serum for 1 h and incubated with primary antibodies at 4 °C overnight. After washing with PBS, biotinylated anti-goat IgG (Vector Laboratories, Inc., Burlingame, CA, USA) was added for 40 min. After washing with PBS, avidin–biotin complex (1:200, Vector Laboratories Co., USA) was added for 60 min. After washing with PBS, the sections were developed with freshly prepared 3,3′-diaminobenzidine (DAB, Vector Laboratories Co., USA). The sections were dried overnight at room temperature and then mounted with a mounting medium (Thermo Electron Co., USA).

### 2.8. Double Immunofluorescence Staining

The sections were blocked with 5% normal horse serum and incubated overnight at 4 °C with primary antibodies. After washing with PBS, the sections were incubated with secondary antibodies (donkey anti-goat secondary antibody conjugated to Dylight™ 488 and donkey anti-goat secondary antibody conjugated to Dylight™ 594 (1:500, Jackson ImmunoResearch Laboratories, USA). Afterwards, the sections were washed again with PBS and mounted with 3% n-propyl gallate and 50% glycerol in PBS. The specimens were viewed under a fluorescence microscope (Nikon, Tokyo, Japan).

### 2.9. Image Analysis

The sections were imaged with a microscope (Nikon, Japan) connected to a digital camera and computer. The number of G-CSF-immunoreactive cells and the total number of cells in the DRG at different time points were calculated and quantified with Image-Pro Plus 6.0 software (Media Cybernetics Inc., Silver Spring, MD, USA). At least 10 sections from each animal were assessed.

### 2.10. Blood Sample Collection and Analysis

The rats were anesthetized with 1.5% isoflurane (Panion & BF Biotech Inc., Taoyuan, Taiwan) and blood samples were collected on day 1, day 7 and day 14 after treatment. The blood samples were collected from the tail vein of the rats and placed into EDTA-containing tubes. Samples were taken for a complete blood count, including white blood cells and neutrophils. The samples were analyzed within 1 h of collection, using the ADVIA 2120 analyzer (Bayer HealthCare, Diagnostics Division, Tarrytown, NY, USA).

### 2.11. Statistical Analysis

Statistical analyses were performed, using the Statistical Program for Social Sciences (SPSS) software (version 22.0; SPSS Inc., Chicago, IL, USA). Quantitative data are presented as the mean ± standard derivation (SD). Data were tested for normality, using the Shapiro–Wilk test. For immunohistochemistry, the quantitative data were statistically analyzed using Student's t-test. Differences in pain behavior were analyzed using one-way analysis of variance with Bonferroni’s post hoc test to determine the statistical significance. Differences were considered significant at ^#^ *p* < 0.05 and highly statistically significant at ^##^ *p* < 0.01, ^###^ *p* < 0.001.

## 3. Results

The Shapiro–Wilk data indicated that all data were normally distributed (*p* values ranging from 0.183 and 0.905).

### 3.1. G-CSFR Is Expressed in Small- and Medium-Sized DRG Neurons

**Figure 1:**
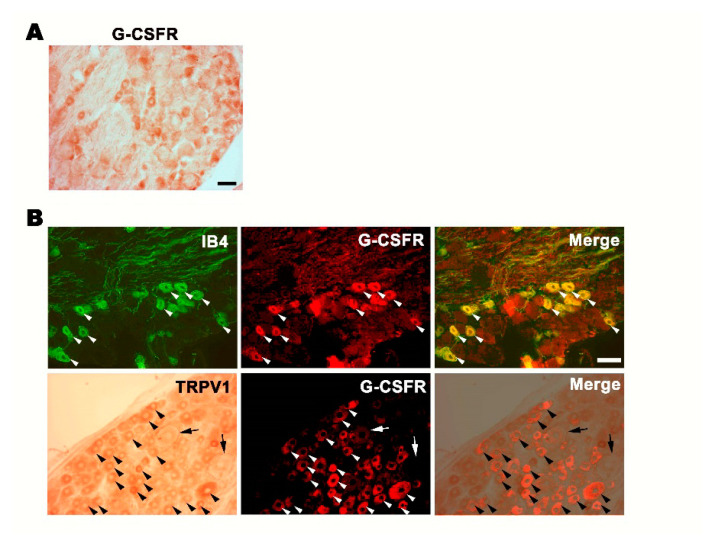
G-CSFR expressed in small- and medium-diameter DRG neurons. (**A**) G-CSFR immunohistochemistry. Bar = 50 μm. (**B**) G-CSFR was expressed in small- and medium-diameter DRG neurons and nociceptive neurons in the DRG. DRG sections were double stained with IB4 and G-CSFR antibodies or TRPV1 and G-CSFR antibodies. Note that G-CSFR was co-expressed with nociceptive TRPV1 (arrowhead), but not in large-diameter DRG (arrow). Bar = 30 μm.

We first examined whether G-CSFR is present in the DRG. We collected the DRG from wildtype rats and performed immunostaining for G-CSFR. As shown in Figure 1A, we observed positive immunoreactivity for G-CSFR and suspected that it was present in small- and medium-sized DRG neurons but not in large-diameter DRG neurons. To further determine whether G-CSFR was expressed in small- and medium-sized DRGs, we performed double immunostaining with isolectin B4 (IB4, a marker of small- and medium-sized DRG neurons), anti-G-CSFR or anti-TRPV1 (markers of nociceptive neurons) and G-CSFR, respectively. As shown in Figure 1B, we found that G-CSFR was co-expressed in IB4-positive and TRPV1-positive DRG neurons (Figure 1B, arrowheads), but not in large-sized DRG neurons (Figure 1B, arrowheads), indicating that G-CSFR was expressed in small- and medium-sized nociceptive DRG neurons.

### 3.2. Endogenous G-CSF Is Expressed in Small and Medium-Size DRG Neurons and Satellite Cells

**Figure 2:**
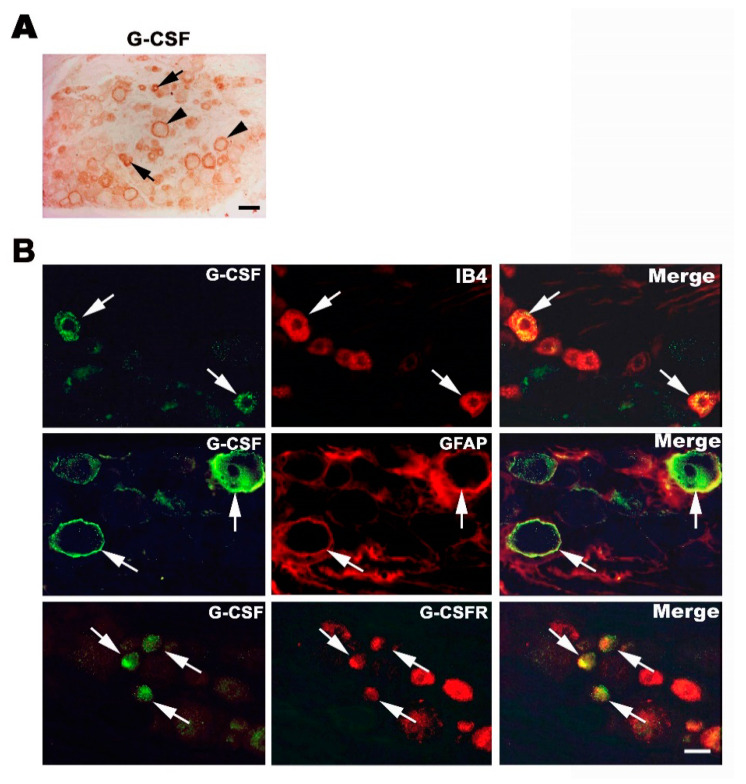
Expression of G-CSF in the DRG. (**A**) G-CSF immunohistochemistry. Note that G-CSF was expressed in small- and medium-diameter DRG neurons (arrow) and satellite cells around large-diameter DRG neurons (arrowhead). Bar = 50 μm. (**B**) Characteristics of the cell types expressing G-CSF in the DRG. DRG sections were double immunostained with anti-G-CSF and IB4, anti-G-CSF and anti-GFAP or anti-G-CSF and anti-G-CSFR antibodies. Co-stained cells are indicated by arrows. Bar = 30 μm.

We then tested whether G-CSF is expressed in the DRG. We collected the DRG from normal rats and performed immunostaining for G-CSF. As shown in Figure 2A, G-CSF was expressed, presumably in small- and medium-sized DRG neurons (Figure 2A, arrow). In addition, anti-G-CSF staining was also found around large-diameter DRG neurons (Figure 2A, arrowhead). We aimed to determine the identity of cells expressing G-CSF. As shown in Figure 2B, G-CSF was co-expressed with IB4, indicating that G-CSF was expressed in small- and medium-sized DRG neurons. We also confirmed that in the DRG, G-CSF was co-expressed with GFAP, showing that the circular staining pattern seen in the immunohistochemical staining image corresponded to a satellite glial cell. Furthermore, G-CSF was co-expressed in G-CSFR-positive DRG neurons (Figure 2B), indicating the autocrine or paracrine fashion of G-CSF physiological functions.

### 3.3. Changes in G-CSF Expression in the DRG in Rats with Partial Sciatic Nerve Transection (PST)

**Figure 3:**
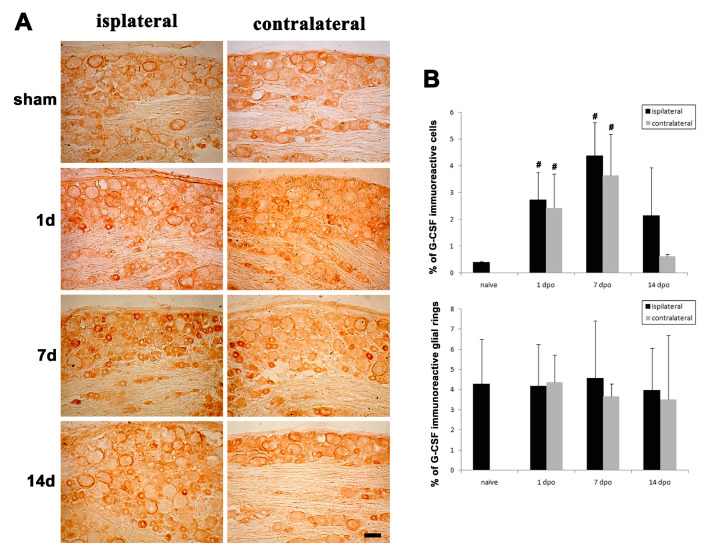
Changes in endogenous G-CSF expression after sciatic nerve injury. (**A**) Rats were sacrificed at 1, 7 and 14 days after PST, and DRG tissues were collected from ipsilateral and contralateral sides and subjected to immunostaining for G-CSF, which was compared between the PST group and the sham group. Bar = 50 μm. (**B**) Quantification of the number of G-CSF-positive DRG neurons on the ipsilateral and contralateral sides in rats with PST. The data are presented as the mean ± SD, ^#^ *p* < 0.05.

We confirmed that endogenous G-CSF was expressed in the DRG and then assessed whether the expression of G-CSF was altered in rats with PST. G-CSF expression in the DRG was compared at 1, 7 and 14 days after surgery. Immunohistochemical staining for G-CSF showed that G-CSF was expressed in small- and medium-sized DRGs and satellite glial cells in the sham group (Figure 3A). Beginning on the first day after PST, intensive G-CSF immunoreactivity was observed in the L4/5 DRG on the ipsilateral side (lesion side) and contralateral side and expressed on the small- and medium-sized DRG neurons. The quantitative results showed that the number of G-CSF-positive DRG neurons on the ipsilateral side and contralateral side was significantly increased 1 day after surgery, compared with the sham-operated rats (^#^ *p* < 0.05), which peaked at 7 days after surgery (^#^ *p* < 0.05) and was decreased at 14 days after surgery. At 14 days after surgery, there was no significant difference between the number of cells expressing G-CSF on the ipsilateral and contralateral sides in the PST rats, compared with the sham-operated rats (Figure 3A,B). However, the number of satellite glial cells showing G-CSF immunoreactivity was not significantly altered at different time points after surgery (Figure 3A,B).

### 3.4. G-CSF Induces Pain Behavior

**Figure 4:**
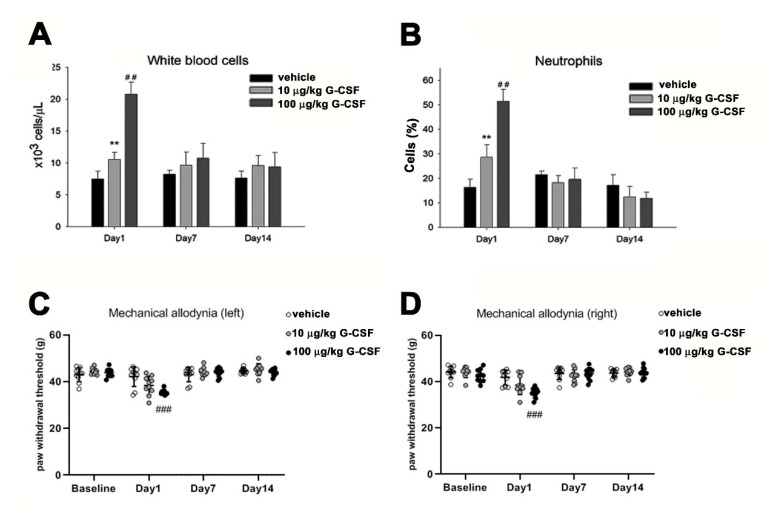
Effect of exogenous G-CSF on wildtype rat behavior and blood cell numbers. (**A**) Histograms showing the results of white blood cell analysis (×10^3^ cells/µL) in the vehicle (normal saline solution) group, G-CSF (10 μg/kg) group and G-CSF (100 μg/kg) group. White blood cell analysis was performed with a Bayer ADVIA 2120 hematology analyzer. ** *p* < 0.01, G-CSF (10 μg/kg) group compared with the vehicle group; ^##^ *p* < 0.01, G-CSF (100 μg/kg) group compared with the vehicle group. (**B**) Histograms showing the results of neutrophil analysis in the 3 groups of rats. Neutrophil analysis was performed with a Bayer ADVIA 2120 hematology analyzer. ** *p* < 0.01, G-CSF (10 μg/Kg) group compared with the vehicle group; ^##^ *p* < 0.01, G-CSF (100 μg/Kg) group compared with the vehicle group. Left (**C**) and right (**D**) paw withdrawal test for mechanical allodynia. Left and right feet of pain behavioral responses of the 3 groups of rats (n = 10 per group). Mechanical allodynia of both feet was assessed using a DPA. ^###^ *p* < 0.001, G-CSF (100 μg/Kg) group, compared with the vehicle group.

To further elucidate the role of G-CSF in pain transmission, we tested whether the administration of different doses of exogenous G-CSF affects the pain behavior of rats. Clinically, G-CSF can cause white blood cells to proliferate. We intravenously injected low-dose (10 μg/kg) and high-dose (100 μg/kg) G-CSF. At both doses, G-CSF induced the proliferation of white blood cells and neutrophils within one day (^##^ *p* < 0.01). However, 7 and 14 days after G-CSF injection, the numbers of white blood cells and neutrophils had returned to normal (Figure 4A,B). In Figure 4C,D, the mechanical pain test of both feet showed that wild-type rats developed symptoms of allodynia 24 h after intravenous (i.v.) injection of 100 ìg/kg G-CSF. One-way ANOVA indicated significant differences between the means of the three groups in the left side (F(_2, 27_) = 9.655, *p* < 0.001) and right side (F(_2, 27_) = 11.022, *p* < 0.001). Bonferroni post hoc analysis further showed that significant differences were observed between 100 ìg/kg G-CSF and vehicle groups (^###^ *p* < 0.001) in both sides. However, there was no difference between 10ug/kg G-CSF and vehicle groups. G-CSF (100 ìg/kg)-induced mechanical allodynia subsided with time.

### 3.5. G-CSF Enhances Mechanical Allodynia Following PST

**Figure 5:**
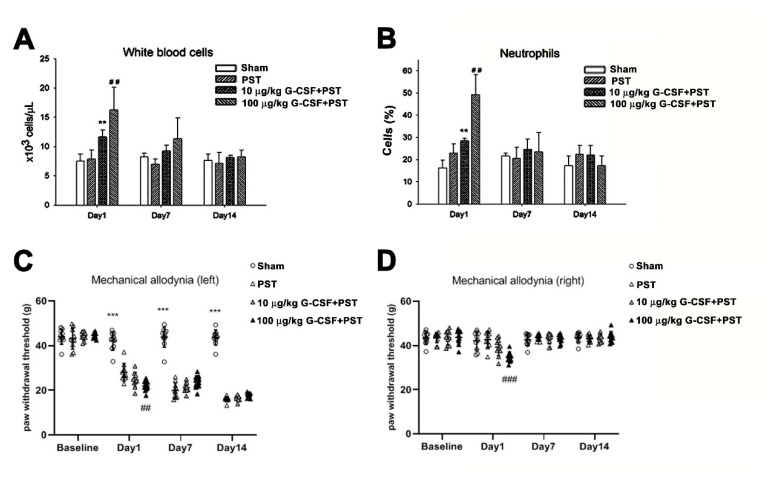
Effect of exogenous G-CSF on PST-induced rat behavior and blood analysis. (**A**) Histograms showing the results of white blood cell analysis (×10^3^ cells/µL) in the sham group, PST group, G-CSF (10 μg/kg) +PST group and G-CSF (100 μg/kg) + PST group. White blood cell analysis was performed with a Bayer ADVIA 2120 hematology analyzer. ** *p* < 0.01, G-CSF (10 μg/kg) + PST group compared with the PST group; ^##^ *p* < 0.01, G-CSF (100 μg/kg) + PST group compared with the PST group. (**B**) Histograms showing the results of neutrophil analysis in the 4 groups of rats. Neutrophil analysis was performed with a Bayer ADVIA 2120 hematology analyzer. ** *p* < 0.01, G-CSF (10 μg/kg) + PST group compared with the PST group; ^##^ *p* < 0.01, G-CSF (100 μg/kg) + PST group compared with the PST group. Left (**C**) and right (**D**) paw withdrawal test for mechanical allodynia. Pain behavioral responses of the 4 groups rats (n = 10 per group). Mechanical allodynia was assessed using a DPA. (C) Pain behavior on the left side, ^##^ *p* < 0.01, G-CSF (100 μg/kg) + PST group, compared with the PST group. *** *p* < 0.001, PST and G-CSF+PST groups, compared with sham group. (D) Pain behavior on the right side, ^###^ *p* < 0.001, G-CSF (100 μg/kg) + PST group compared with the PST group.

We then examined the effect of G-CSF on the nociceptive response in rats with PST. Before examination, we tested the effect of G-CSF on changes in the number of white blood cells in rats with PST. As shown in Figure 5, rats with PST did not exhibit an increased numbers of white blood cells and neutrophils on days 1, 7 and 14 after surgery (Figure 5A,B). However, administration of low-dose (10 ìg/kg) and high-dose (100 ìg/kg) G-CSF induced significant proliferation of white blood cells and neutrophils one day after surgery, but on the 7th and 14th days after surgery, the number of white blood cells and the number of neutrophils had returned to normal (Figure 5A,B). In Figure 5C,D, PST rats exhibited different behavioral responses to pain in the lesion side (left) and no lesion (right) side. On the lesion side (left), the left hind paw of PST or G-CSF+PST rats exhibited mechanical allodynia symptoms 1, 7 and 14 days after surgery, compared to the sham group. One-way ANOVA indicated significant differences on day 1 after surgery (F(_3, 36_)= 65.740, *** *p* < 0.001), 7 day (F(_3, 36_) = 110.969, *** *p* < 0.001) and 14 day (F(_3, 36_) = 464.080, *** *p* < 0.001). Bonferroni post hoc analysis further showed that high-dose (100 ìg/kg), but not low-dose (10 ìg/kg), G-CSF administration aggravated the symptoms of mechanical allodynia in rats with PST on day 1 after surgery (^##^ *p* < 0.01). However, 7 and 14 days after G-CSF administration, the rats exhibited an equivalent degree of allodynia as rats with PST (Figure 5C).

On the no lesion side (right), the right hind paw of the PST rats did not develop mechanical allodynia during experiments performed after surgery. The one-way ANOVA only indicated a significant difference (F(_3, 36_) = 10.249, *p* < 0.001) on day 1 after surgery. Bonferroni post hoc analysis further showed that high-dose rather than low-dose G-CSF administration to PST rats exhibited mechanical allodynia (^###^ *p* < 0.001). This effect faded over time (Figure 5D).

## 4. Discussion

In this study, we demonstrated that endogenous G-CSF is expressed in small- and medium-sized DRGs and satellite glial cells. In a rat model of PST, the expression of endogenous G-CSF in nociceptive neurons increased, and endogenous G-CSF expression was maintained 7 days after surgery. Using double immunofluorescence staining, we also confirmed that G-CSF and G-CSFR were co-expressed in nociceptive neurons. This suggests that G-CSF may act through autocrine or paracrine mechanisms. To clarify the role of G-CSF in pain transmission, exogenous G-CSF was administered to normal rats and rats with PST. The results showed that G-CSF can cause pain sensitivity under normal circumstances. In addition, it may aggravate neuropathic pain symptoms in rats with PST.

This study confirmed that under normal conditions, a small amount of G-CSF was expressed in nociceptive neurons and satellite glial cells in the DRG. After PST, the G-CSF expression level was increased in some nociceptive neurons; the number of G-CSF-immunoreactive cells was increased on the first day after surgery, continued to increase until 7 days postoperatively, and tended to be decreased within 14 days after surgery. Compared with the contralateral side, the lesion side exhibited more pain mechanisms, such as injury discharge, redistribution of sodium channels, endoneurial upregulation of neurotrophic factors and proinflammatory cytokines [30], perhaps synergistically with G-CSF to occur pain behavior. It is known that during the course of neuropathic pain, a large amount of growth factors and cytokines are expressed in the DRG [34]. The gene expression of the cytokines TNF-α and IL-1β, which are related to the production of G-CSF, greatly increased after 1 day [35]. Although it was not confirmed whether endogenous G-CSF in the DRG is directly induced by TNF-α and IL-1β, this study confirmed that PST can induce G-CSF expression in the DRG, suggesting that G-CSF is involved in the development of early neuropathic pain.

It is known that neurotrophic factors are abundantly expressed in the early stages after nerve injury [36,37]. Previous studies have confirmed that in different rat models of ischemic stroke, the gene expression level of G-CSF in the cortex increases by more than a hundred times within 6 h after surgery [18,38]. Similar results were obtained by immunohistochemical staining [39,40]. This study confirmed that G-CSF expression is elevated in nociceptive afferent neurons after sciatic nerve injury and is co-expressed with G-CSFR. G-CSF was found to act on tumor cells and nerve cells through autocrine and paracrine mechanisms [41], indicating that G-CSF might regulate afferent neurons through autocrine or paracrine mechanisms in the nervous system. DRG and satellite glial cells communicate with each other via paracrine signaling [42]. It is generally known that substance P, ATP and CGRP secreted by sensory neurons can affect the excitability of satellite glial cells [43], but it is not yet clear which molecules drive the activation of satellite glial cells under pathological conditions. This experiment confirmed that satellite glial cells express G-CSF under normal physiological conditions. However, the number of satellite glial cells exhibiting G-CSF immunoreactivity in the PST model did not change significantly, indicating that G-CSF secreted by satellite glial cells may not participate in the development of neuropathic pain. Therefore, more research is needed on the physiological significance of G-CSF secreted by satellite glial cells in the DRG.

Many studies have reported that G-CSF is neuroprotective, promotes neurogenesis, and alters neuroplasticity [17]. Schweizerhof et al. demonstrated that G-CSF secreted by bone cancer cells causes peripheral neurosensitization and increases the expression of TRPV1 and Nav1.8 via the JAK/STAT and ERK pathways [44]. G-CSF has been shown to increase TRPV1 and G-CSFR expression via STAT3 signaling in neuronal cultures [45]. Although the mechanism by which G-CSF affects the development of neuropathic pain was not confirmed in this study, behavioral assessments confirmed that subcutaneous injection of exogenous G-CSF induces the development of neuropathic pain symptoms. However, whether G-CSF alters the expression of ion channels involved in abnormal pain, such as TRPA1 and TRPV4, was not reported in this study; more experimental evidence is needed.

A previous study reported that in G-CSF knockout mice and mice lacking G-CSF in the CA3 and dentate gyrus of the hippocampus, the density of NMDA receptors was significantly reduced [46], indicating that G-CSF may regulate NMDA receptors. Therefore, we infer that administration of low-dose exogenous G-CSF may increase NMDA receptor expression in postsynaptic neurons, resulting in central neurosensitization. Therefore, these studies suggest that the treatment of neurological disorders with G-CSF may lead to unintended pain.

Based on these results, endogenous G-CSF is expressed in nociceptive neurons. Additionally, nerve damage leads to increased expression of G-CSF, while exogenous administration of G-CSF increases nociceptive responses, suggesting that G-CSF may play an important role in pain modulation in nociceptive neurons.

## 5. Conclusions

Endogenous G-CSF is expressed in small- and medium-sized DRG neurons and satellite glial cells. After sciatic nerve injury, the endogenous expression of G-CSF in small- and medium-size DRG neurons was increased. Treatment with exogenous G-CSF exacerbated neuropathic pain. Therefore, G-CSF may directly activate sensory neurons and contribute to nociceptive signaling.

## Data Availability

Not applicable.
